# Supplementary material for: Prenatal Diagnosis of Right-Sided Congenital Ventricular Diverticulum (CVD) Assisted by Spatiotemporal Image Correlation (STIC) Acquisition and the Speckle-Tracking Technique to Assess Fetal Cardiac Function: A Case Report
Source: Diagnostics (Basel). 2022 Oct 8;12(10):2438. doi: 10.3390/diagnostics12102438 (PMC9600030; doi:10.3390/diagnostics12102438)
Supplement: Supplementary file 1 [file diagnostics-12-02438-s001.zip › supplementary figure S1.pdf]

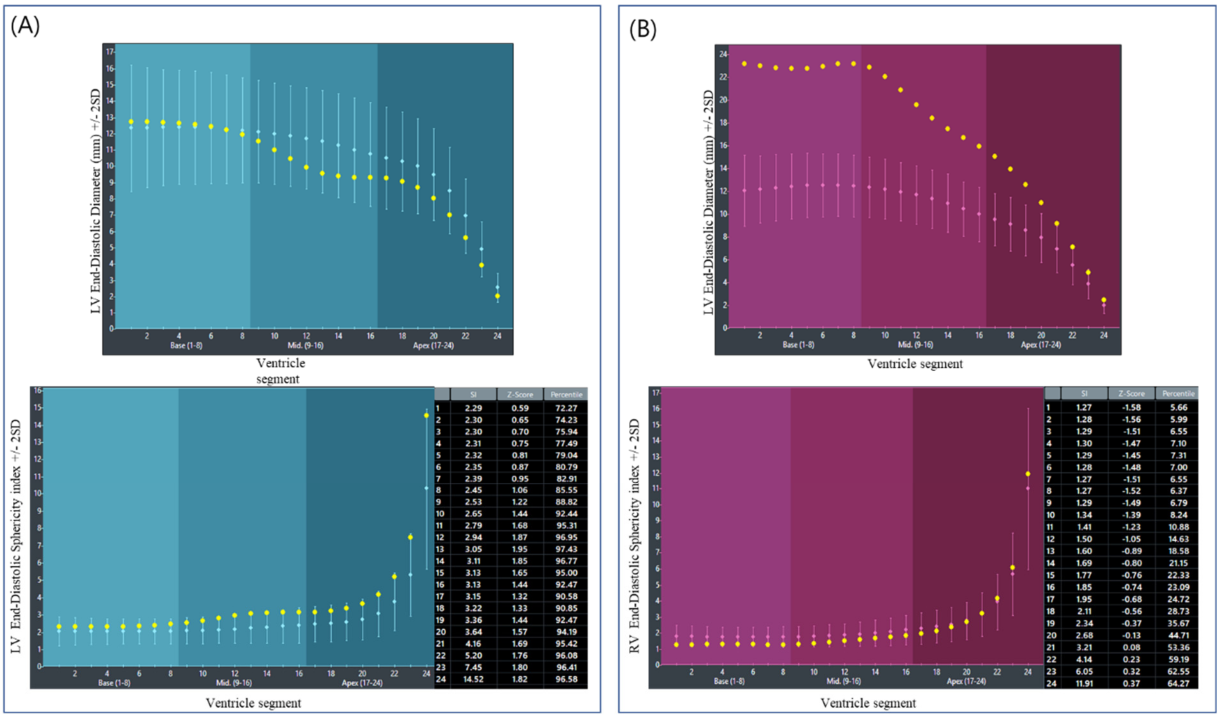


LV, left ventricle; Rt, right; RV, right ventricle; SD; standard deviation.

Supplementary Figure S1. Twenty-four-segment ventricular analysis and the 24-segment sphericity index at a GA $33^{+5}$ weeks

(A) LV geometry of the fetus with CVD. The upper graph shows the results of the 24-segment ventricular analysis. The lower graph shows the results of the 24-segment sphericity index analysis.

(B) RV geometry of the fetus with CVD. The upper graph shows the results of the 24-segment ventricular analysis. The lower graph shows the results of the 24-segment sphericity index analysis. The RV was wider than the reference value and was particularly prominent at the base (1-8) and mid (9-16) regions, the widths of which were out of range(C) 24-segment sphericity index of RV. RV has was wider than reference and was particularly prominent in the base (1-8) and mid (9-16), which was out of range. LV; left ventricle, Rt; right, RV; right ventricle, SD; standard deviation.
