# Supplementary material for: Prenatal Diagnosis of Right-Sided Congenital Ventricular Diverticulum (CVD) Assisted by Spatiotemporal Image Correlation (STIC) Acquisition and the Speckle-Tracking Technique to Assess Fetal Cardiac Function: A Case Report
Source: Diagnostics (Basel). 2022 Oct 8;12(10):2438. doi: 10.3390/diagnostics12102438 (PMC9600030; doi:10.3390/diagnostics12102438)
Supplement: Supplementary file 1 [file diagnostics-12-02438-s001.zip › supplementary figure S2.pdf]

Supplementary figure S2.

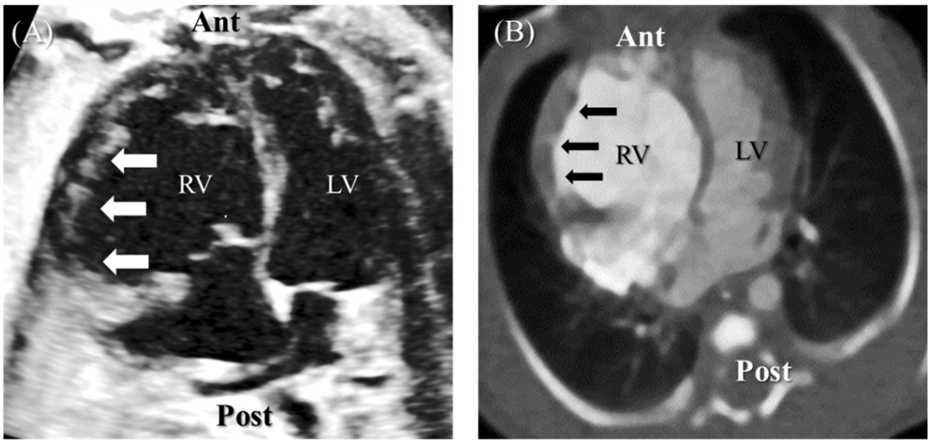

Ant, anterior; Lt, left; LV, left ventricle; Post, posterior; Rt, right; RV, right ventricle

(A) At 35+2 weeks GA, fetal echocardiography of the fetus with CVD. The outpouching ventricular wall (white arrowhead) was observed.

(B) Chest computed tomography of the baby with CVD. It illustrated a heart axial plane similar to the antenatal four-chamber view and showed the outpouching ventricular wall (black arrowhead)
